# Supplementary material for: Clonal effects of the Ras oncogene revealed by somatic mutagenesis in a Drosophila cancer model
Source: bioRxiv. 2025 May 10:2025.05.08.652841. Preprint. [Version 1] doi: 10.1101/2025.05.08.652841 (PMC12248059; doi:10.1101/2025.05.08.652841)

bioRxiv preprint doi: https://doi.org/10.1101/2025.05.08.652841; this version posted May 10, 2025. The copyright holder for this preprint (which was not certified by peer review) is the author/funder, who has granted bioRxiv a license to display the preprint in perpetuity. It is made available under aCC-BY 4.0 International license.

Figure S1. Comparison between human K-Ras, N-Ras, H-Ras and *Drosophila* Ras85D proteins.

Oncogenic mutation hot spots (G12, G13 and Q61) and each protein domain are highlighted. The bold characters indicate the conserved amino acid residues between three Ras proteins.

Figure S2. Tissue-specific accumulation of *K-Ras*, *N-Ras*, and *H-Ras* oncogenic mutations.

The numbers in parentheses indicate sample sizes.

Figure S3. Generation of *Ras85D* inducible alleles.

(A) The schematic illustration depicts the process of establishing inducible *Ras85D* alleles. **(B-S)** Different *Ras85D* alleles were induced in the wing pouch area using *nubbin-Gal4* and *UAS-FLP*. The activation of the MAPK pathway was visualized through anti-dp-ERK staining (*magenta*) in the wing discs, both before **(B**, **E**, **H**, **K**, **N**, **Q)** and after **(C**, **D**, **F**, **G**, **I**, **J**, **L**, **M**, **O**, **P**, **R**, **S)** the allele switch induction**.** Loss of GFP (*green*) signals indicates the population of cells switched from *wild-type* to desired *Ras85D* alleles. Scale bar: 100 μm.

Figure S4. *Ras85D* oncogenic mutation triggers a cell-autonomous activation of MAPK signaling.

(A-I) Small clones of cells expressing *wild-type Ras85D* (**A**-**C**), *G12V* (**D**-**F**), and *Q61H* (**G**-**I**) were randomly induced in the wing disc using *hs-FLP*. dp-ERK staining demonstrated a cell-autonomous MAPK activation in the clones of cells expressing *Ras85D* oncogenic mutations marked by loss of GFP (*green*). 50 μm in **A**; 20 μm in **B.**

bioRxiv preprint doi: https://doi.org/10.1101/2025.05.08.652841; this version posted May 10, 2025. The copyright holder for this preprint (which was not certified by peer review) is the author/funder, who has granted bioRxiv a license to display the preprint in perpetuity. It is made available under aCC-BY 4.0 International license.

Figure S5. *Ras85D* oncogenic *G12D*, *G13D*, and *G13R* mutations lead to the cyst formation the wing disc.
(A-C) *G12D* (**A**), *G13D* (**B**), and *G13R* (**C**) oncogenic mutations induced cysts in the wing discs. The locations of mutant clones are indicated by the loss of GFP signals (*green*). Scale bar: 50 μm.

Figure S6. *Ras Q61H* mutant cells differentiate into enteroendocrine cells and enterocytes, maintaining the integrity of the epithelial structure.
(**A**-**D**) *Ras Q61H* mutant cells in the *esg-Gal4/+; tub-Gal80*^{ts}*, UAS-FLP/Ras85D*$^{GFP>>Q61H}$ midguts generate Prospero positive enteroendocrine cells (Pros, *magenta* in **A**) and enterocytes expressing Pdm1 (*magenta* in **C**). (**E-I**) Inducing *Ras* Q61H mutations using *esg*^{ts}*FLP* increased the intestinal stem cells detected by anti-Delta antibody compared to the control midguts (Dl, *magenta* in **E** and **G**). (I) Quantification of intestinal stem cell numbers. Two-sided Student's *t-test*: *$P < 0.05$. (**J**-**K**) Intestinal epithelial structures of the control *esg-Gal4/+; tub-Gal80ts, UAS-FLP/Ras85D*$^{GFP>>wt}$ and *esg-Gal4/+; tub-Gal80ts, UAS-FLP/Ras85D*$^{GFP>>Q61H}$ midguts visualized by anti-Dlg and Sir-Actin staining. Scale bars: 10 μm.

bioRxiv preprint doi: https://doi.org/10.1101/2025.05.08.652841; this version posted May 10, 2025. The copyright holder for this preprint (which was not certified by peer review) is the author/funder, who has granted bioRxiv a license to display the preprint in perpetuity. It is made available under aCC-BY 4.0 International license.

**Primer**	**Primer sequence (5' to 3')**	**Purpose**
1	CTTCGAGGTGAATTTGCTAATTCGG	*Ras85D* sgRNA construct 1
2	AAACCCGAATTAGCAAATTCACCTC	*Ras85D* sgRNA construct 1
3	CTTCGACAAGACAGGAGACTTTAGA	*Ras85D* sgRNA construct 2
4	AAACTCTAAAGTCTCCTGTCTTGTC	*Ras85D* sgRNA construct 2
5	CCGGGTACCGAGCTCGAA	*Ras85D allele switch founder* donor *pHSG298* fragment forward primer
6	GGATCCTCTAGAGTCGACCTG	*Ras85D allele switch founder* donor *pHSG298* fragment reverse primer
7	CCTGCAGGTCGACTCTAGAGGATCCCCAC AAACTTGTCCACCAG	*Ras85D allele switch founder* donor left arm homology fragment forward primer
8	GAATAGGAACTTCCGGCGGCGTTGTTTTT AGCCCTTTTC	*Ras85D allele switch founder* donor left arm homology fragment reverse primer
9	AACAACGCCGCCGGAAGTTCCTATTCCGA AGTTCCTATTCTCTAGAAAGTATAGGAACT TCAATTAGCAAATTCACCTAGTTAACAACT C	*Ras85D allele switch founder* donor *5' FRT_Ras85D 5' UTR*_coding exon_*3' UTR* fragment forward primer
10	CTACTCCGAATTCAGATGGAAAAATAGAAT CCACAAAAGCAAAACTATGGAATAAC	*Ras85D allele switch founder* donor *5' FRT_Ras85D 5' UTR*_coding exon_*3' UTR* fragment reverse primer
11	TATTTTTCCATCTGAATTCGGAGTAGTGCC CCAACTGGGGTAACCTTTG	*Ras85D* allele switch founder donor *attP_5' loxP_ubi-mCherry* fragment forward primer
12	GACAGGAGACTTTATAACTTCGTATAATGT ATGCTATACGAAGTTATTTAACTTACATACA TACTAGAATTGATCGGCTAAATGGTATGG	*Ras85D* allele switch founder donor *attP_5' loxP_ubi-*

bioRxiv preprint doi: https://doi.org/10.1101/2025.05.08.652841; this version posted May 10, 2025. The copyright holder for this preprint (which was not certified by peer review) is the author/funder, who has granted bioRxiv a license to display the preprint in perpetuity. It is made available under aCC-BY 4.0 International license.

		mCherry fragment reverse primer
13	TATACGAAGTTATAAAGTCTCCTGTCTTGTTGAGCCAAATATTCTATTATTTGAATTTTTGCG	*Ras85D* allele switch founder donor *3' loxP*_right arm homology fragment forward primer
14	TTACGAATTCGAGCTCGGTACCCGGGGAATACTCACAGTCTGTCGTCGTCTC	*Ras85D* allele switch founder donor *3' loxP*_right arm homology fragment reverse primer
15	GGCGGCGTCGACTCGCCAAGCTTGGGCTGCATCAC	*5'* SalI *ubiquitin* promoter-*nuclear eGFP* forward primer
16	GGCGGCACGCGTTTAACTTACATACATACTAGAATTGATCGGC	*ubiquitin* promoter-*nuclear eGFP 3'* MluI reverse primer
17	GGCGGCACGCGTGAAGTTCCTATTCCGAAGTTCCTATTCTCTAGAAAGTATAGGAACTTCAATTAGCAAATTCACCTAGTTAACAACTCG	*Ras85D* allele switch cassette MluI_*3' FRT* forward primer
18	GGCGGCGCGCCATAACTTCGTATAATGTATGCTATACGAAGTTATAGATGGAAAAATAGAATCCACAAAAGCAAAACTATGGAATAAC	*Ras85D* allele switch cassette *3' loxP*_AscI reverse primer
19	GTTGGAGCCGatGGCGTGGGCA	*Ras85D G12D* mutation forward primer
20	GTTGGAGCCGttGGCGTGGGCACGC	*Ras85D G12V* mutation forward primer
22	GACGACCAGTTTGTATTCCGTC	*Ras85D G12D* and *V* mutations reverse primer
23	GGAGCCGGAGatGTGGGCAAGT	*Ras85D G13D* mutation forward primer
24	TGGAGCCGGAcGtGTGGGCAAGT	*Ras85D G13R* mutation forward primer
25	AACGACGACCAGTTTGTATTC	*Ras85D G13D* and *R* mutations reverse primer

bioRxiv preprint doi: https://doi.org/10.1101/2025.05.08.652841; this version posted May 10, 2025. The copyright holder for this preprint (which was not certified by peer review) is the author/funder, who has granted bioRxiv a license to display the preprint in perpetuity. It is made available under aCC-BY 4.0 International license.

26	CCGCCGGCCAcGAGGAGTACT	*Ras85D Q61H* mutation forward primer
27	TGTCCAGGATGTCCAGCAG	*Ras85D Q61H* mutation reverse primer

Supplementary Table. Primers used in this study

Restriction enzyme sites are underlined in primer sequences. Lowercase characters indicate point mutations.

bioRxiv preprint doi: https://doi.org/10.1101/2025.05.08.652841; this version posted May 10, 2025. The copyright holder for this preprint (which was not certified by peer review) is the author/funder, who has granted bioRxiv a license to display the preprint in perpetuity. It is made available under aCC-BY 4.0 International license.

Figure S1. Akiyama and Gibson

```
                     12 13
NRAS       1 MTEYKLVVVGAGGVGKSALTIQLIQNHFVDEYDPTIEDSYRKQVVIDGETCLLDILDTAG 60
KRAS       1 MTEYKLVVVGAGGVGKSALTIQLIQNHFVDEYDPTIEDSYRKQVVIDGETCLLDILDTAG 60
HRAS       1 MTEYKLVVVGAGGVGKSALTIQLIQNHFVDEYDPTIEDSYRKQVVIDGETCLLDILDTAG 60
Ras85D     1 MTEYKLVVVGAGGVGKSALTIQLIQNHFVDEYPDTIEDSYRKQVVIDGETCLLDILDTAG 60
                    10 ___ 17              30 ___ 38                    60
                      P-loop                  Switch-I

             61
NRAS      61 QEEYSAMRDQYMRTGEGFLCVFAINNSKSFADINLYREQIKRVKDSDDVPMVLVGNKCDL 120
KRAS      61 QEEYSAMRDQYMRTGEGFLCVFAINNTKSFEDIHHYREQIKRVKDSEDVPMVLVGNKCDL 120
HRAS      61 QEEYSAMRDQYMRTGEGFLCVFAINNTKSFEDIHQYREQIKRVKDSDDVPMVLVGNKCDL 120
Ras85D    61 QEEYSAMRDQYMRTGEGFLLVFAVNSAKSFEDIGTYREQIKRVKDAEEVPMVLVGNKCDL 120
             _______________ 76
                Switch-II

NRAS     121 PTRTVDTKQAHELAKSYGIPFIETSAKTRQGVEDAFYTLVREIRQYRMKKLNSSDDGTQG 180
KRAS     121 PSRTVDTKQAQDLARSYGIPFIETSAKTRQRVEDAFYTLVREIRQYRLKKISKEEKTPGC 180
HRAS     121 AARTVESRQAQDLARSYGIPYIETSAKTRQGVEDAFYTLVREIRQHKLRKLNPPDESGPG 180
Ras85D   121 ASWNVNNEQAREVAKQYGIPYIETSAKTRMGVDDAFYTLVREIRKDKDNKGRRGRKMNKP 180
                                                        166 _______________
                                                      Hypervariable region

NRAS     181 CMGLPCVVM 189
KRAS     181 VKIKKCIIM 189
HRAS     181 CMSCKCVLS 189
Ras85D   181 NRRFKCKML 189
             ________ 189
```

Figure S2. Akiyama and Gibson

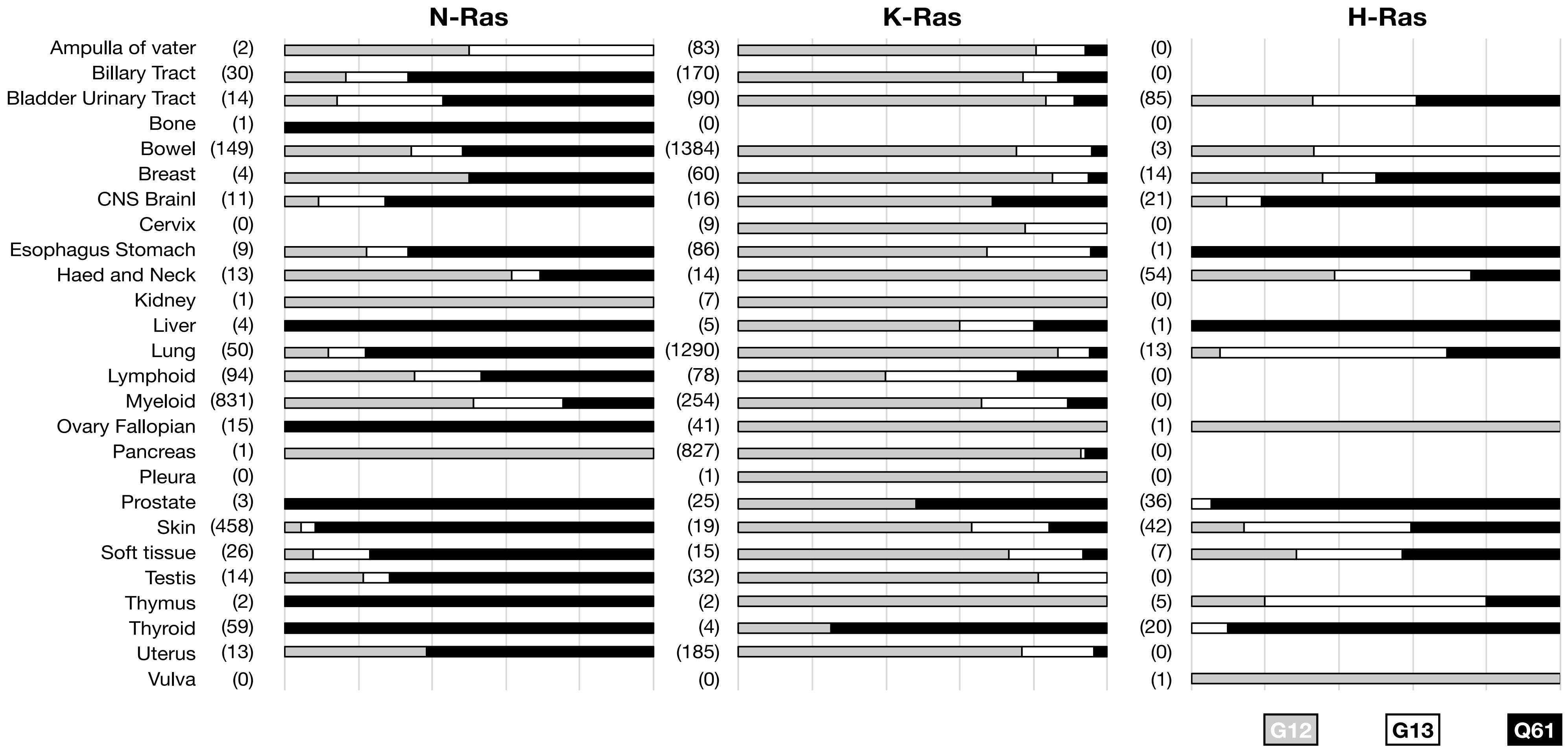

bioRxiv preprint doi: https://doi.org/10.1101/2025.05.08.652841; this version posted May 10, 2025. The copyright holder for this preprint (which was not certified by peer review) is the author/funder, who has granted bioRxiv a license to display the preprint in perpetuity. It is made available under aCC-BY 4.0 International license.

Figure S3. Akiyama and Gibson

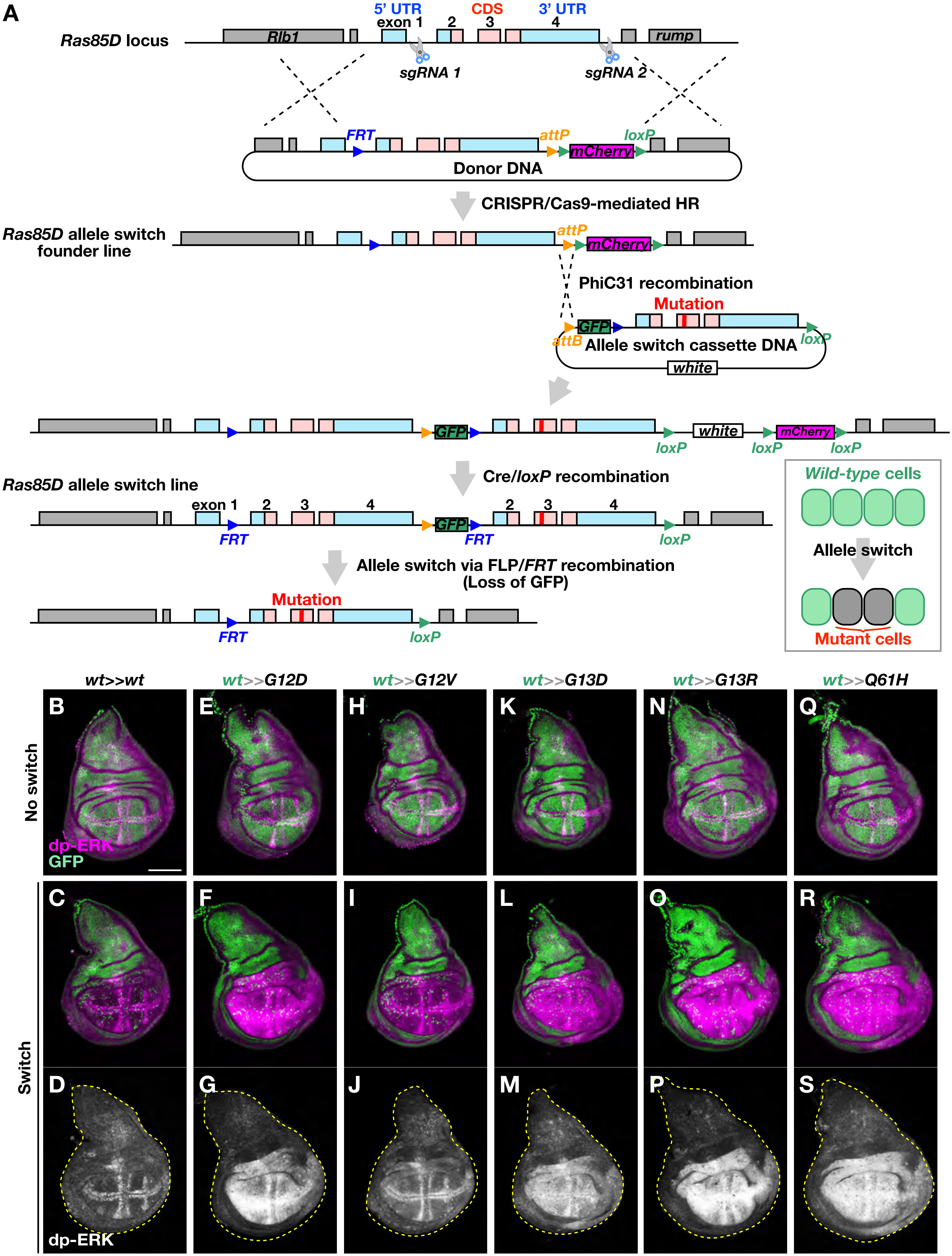

bioRxiv preprint doi: https://doi.org/10.1101/2025.05.08.652841; this version posted May 10, 2025. The copyright holder for this preprint (which was not certified by peer review) is the author/funder, who has granted bioRxiv a license to display the preprint in perpetuity. It is made available under aCC-BY 4.0 International license.

Figure S4. Akiyama and Gibson

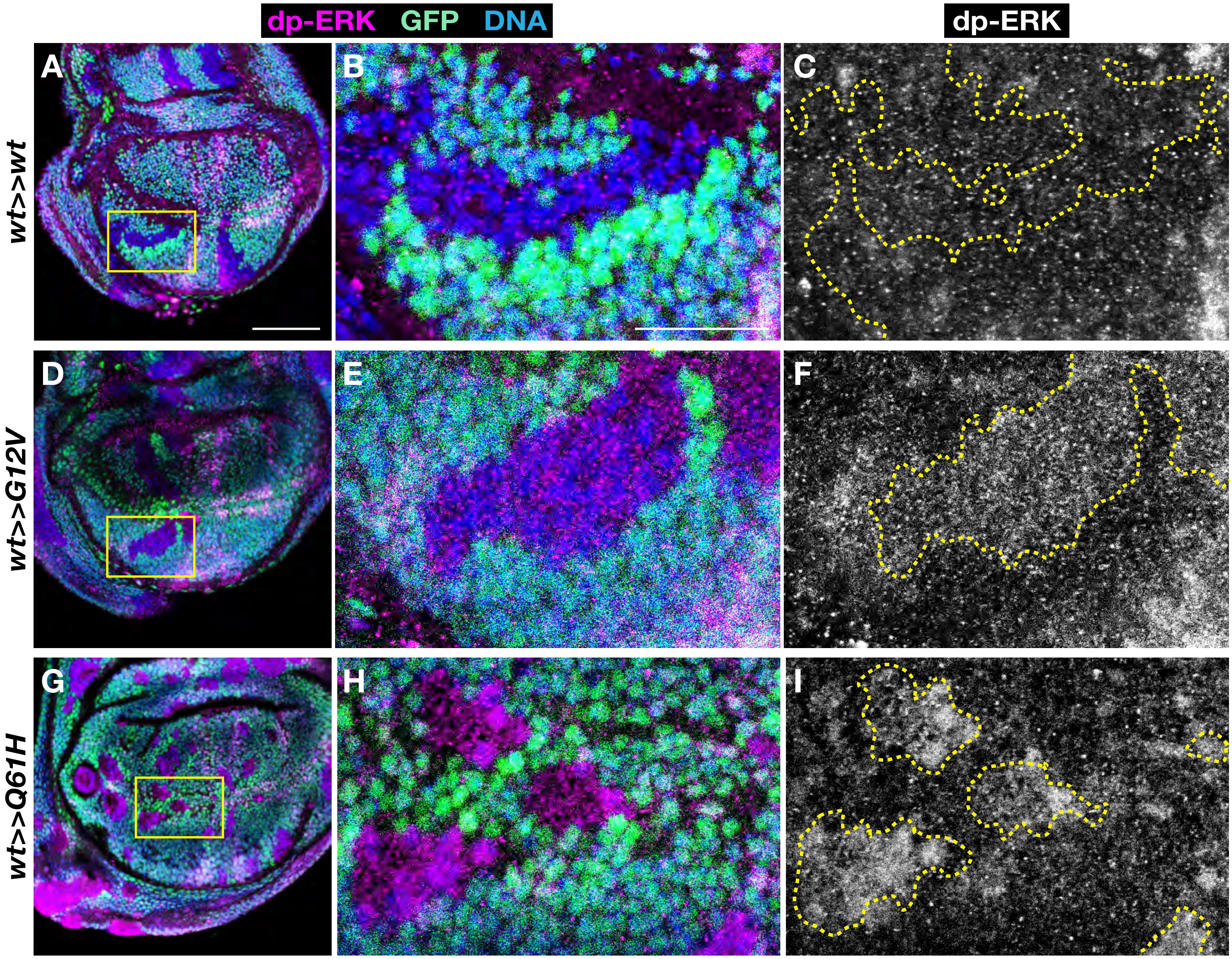

bioRxiv preprint doi: https://doi.org/10.1101/2025.05.08.652841; this version posted May 10, 2025. The copyright holder for this preprint (which was not certified by peer review) is the author/funder, who has granted bioRxiv a license to display the preprint in perpetuity. It is made available under aCC-BY 4.0 International license.

Figure S5. Akiyama and Gibson

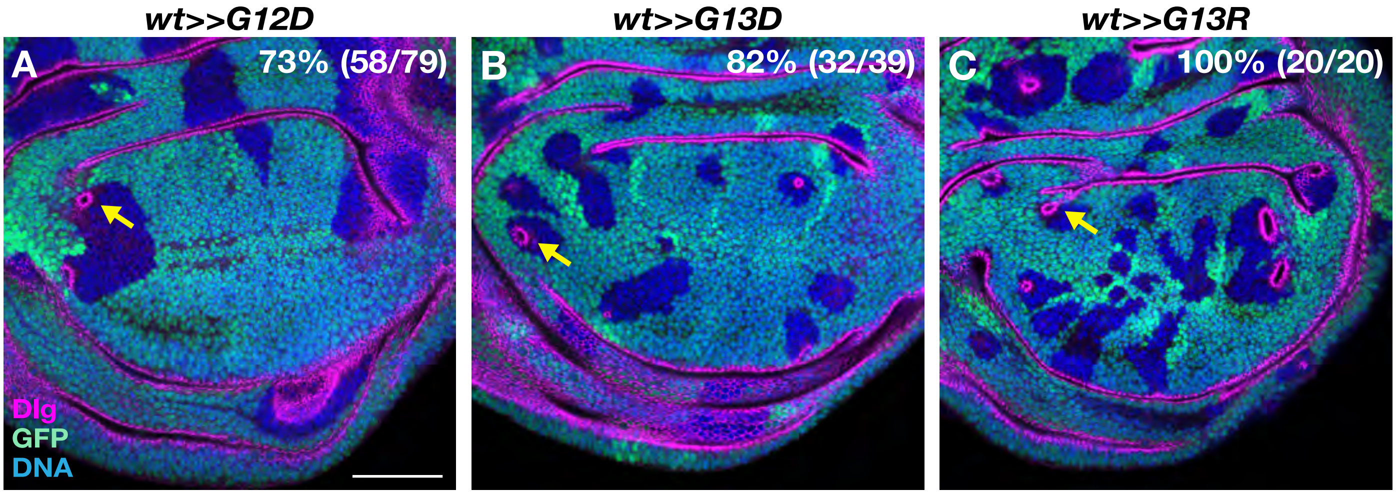

bioRxiv preprint doi: https://doi.org/10.1101/2025.05.08.652841; this version posted May 10, 2025. The copyright holder for this preprint (which was not certified by peer review) is the author/funder, who has granted bioRxiv a license to display the preprint in perpetuity. It is made available under aCC-BY 4.0 International license.

Figure S6. Akiyama and Gibson

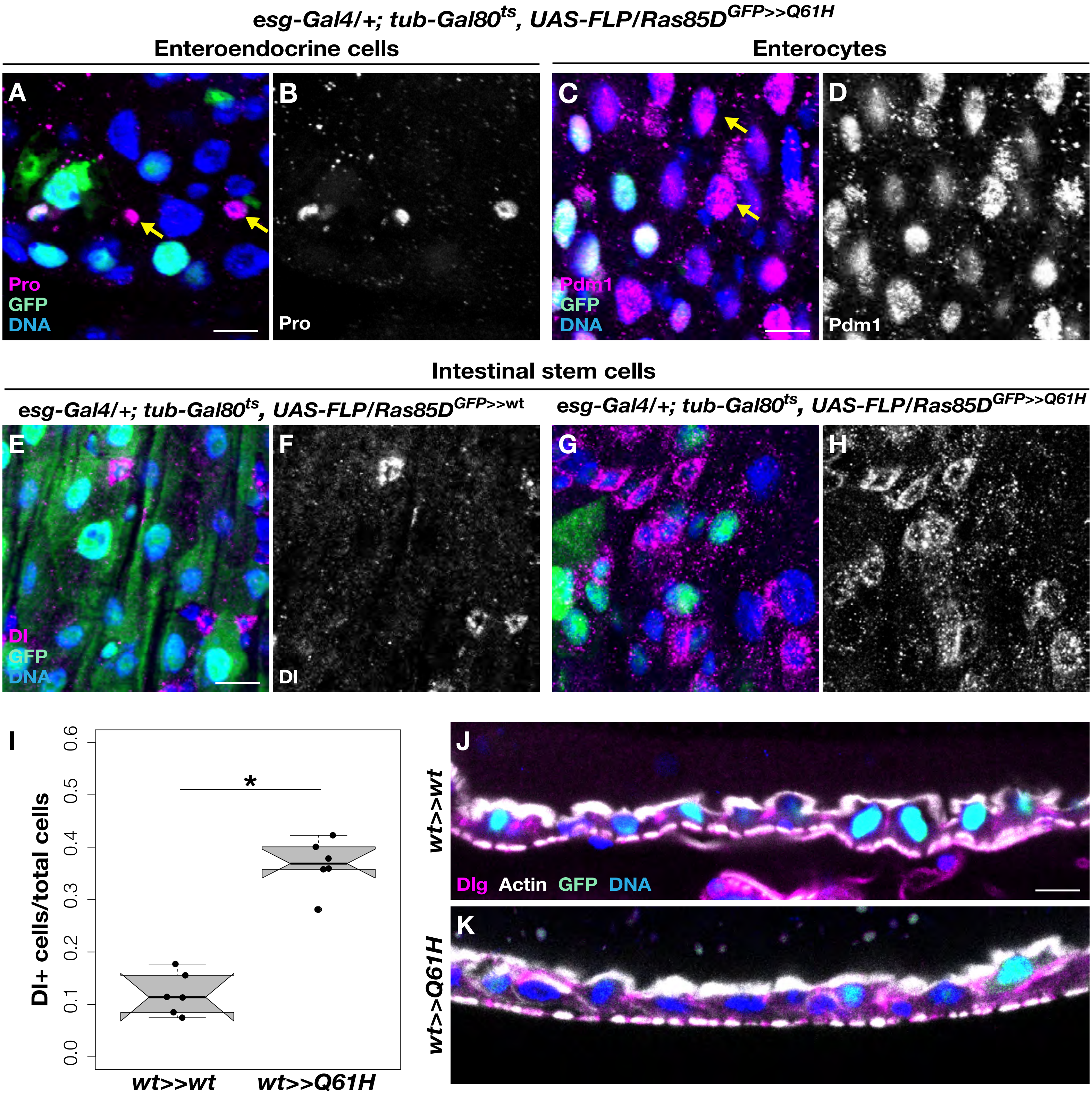

Supplement: 1 [file NIHPP2025.05.08.652841V1-supplement-1.pdf]
